# The Antifungal Activities of Silver Nano-Aggregates Biosynthesized from the Aqueous Extract and the Alkaline Aqueous Fraction of *Rhazya stricta* against Some *Fusarium* Species

**DOI:** 10.3390/nano14010088

**Published:** 2023-12-28

**Authors:** Fatimah Al-Otibi

**Affiliations:** Department of Botany and Microbiology, College of Science, King Saud University, P.O. Box 22452, Riyadh 11495, Saudi Arabia; falotibi@ksu.edu.sa; Tel.: +966-11805597

**Keywords:** *Rhazya stricta*, *Fusarium* spp., UV-vis, FTIR, TEM, DLS, antifungal activities

## Abstract

*Rhazya stricta* is a major medicinal species used in indigenous medicinal herbal medications in South Asia, the Middle East, Iran, and Iraq to treat a variety of ailments. The current study aimed to investigate the antifungal properties of biosynthesized silver nanoparticles (AgNPs) made from *R. stricta* aqueous extract and its alkaline aqueous fraction. Fourier transform infrared spectroscopy (FTIR), UV-vis spectrophotometry, dynamic light scattering (DLS), and transmitted electron microscopy (TEM) were used to characterize AgNPs. The produced extracts and AgNPs were tested for their antifungal efficacy against four *Fusarium* spp. All of the characterization experiments proved the biosynthesis of targeted AgNPs. FTIR showed a wide distribution of hydroxyl, amino, carboxyl, and alkyl functional groups among all preparations. The DLS results showed that the produced Aq-AgNPs and the Alk-AgNPs had an average size of 95.9 nm and 54.04 nm, respectively. On the other hand, TEM results showed that the Aq-AgNPs and Alk-AgNPs had average diameters ranging from 21 to 90 nm and 7.25 to 25.32 nm. Both AgNPs absorbed UV light on average at 405 nm and 415 nm, respectively. Regarding the fungicidal activity, the highest doses of Aq-extract and Aq-AgNPs inhibited the mycelial growth of *F. incarnatum* (19.8%, 87.5%), *F. solani* (28.1%, 72.3%), *F. proliferatum* (37.5%, 75%), and *F. verticillioides* (27.1%, 62.5%), respectively (*p* < 0.001). Interestingly, the Alk-fraction had stronger inhibition than the biosynthesized AgNPs, which resulted in complete inhibition at the doses of 10% and 20% (*p* < 0.001). Furthermore, microscopic analysis demonstrated that both AgNPs caused obvious morphological alterations in the treated organisms when compared to the control. In conclusion, *R. stricta*’s Aq-extract, alkaline fraction, and their biosynthesized AgNPs show substantial antifungal efficacy against several *Fusarium* spp. It is the first study to highlight the prospective biological activities of *R. stricta* Aq-extract and its alkaline fraction against *F. incarnatum*, *F. proliferatum*, and *F. verticillioides*. In addition, it is the first opportunity to deeply investigate the ultrastructural changes induced in the *Fusarium* species treated with *R. stricta* crude Aq-extract and its biosynthesized AgNPs. More studies are required to investigate their biological effect against other *Fusarium* or fungal species.

## 1. Introduction

*Rhazya stricta* Decne is a perennial evergreen dwarf shrub with a smooth central stem and thick semi-erect branches that belongs to the *Apocynaceae* family. It is extensively found in the Middle East and Indian subcontinent [1]. *R. stricta* thrives in depressions with silty and sandy soils, establishing a pure stand at times. It also grows in rocky terrain, hills, plains, wadis, and in the sandy plains of Saudi Arabia [1,2,3]. *R. stricta* leaves are spirally organized, linear-lanceolate, elliptical, or oblanceolate, yellow, and thick when dried. *R. stricta* usually grows in South Asia (Pakistan, India, and Afghanistan) and the Middle East (e.g., Saudi Arabia, Qatar, the United Arab Emirates (UAE), Iran, and Iraq) [1].

*R. stricta* has been used in Saudi traditional medicine to treat ailments such as syphilis, chronic rheumatism, and pain [4]. *R. stricta* is a rich source of alkaloids with a wide range of structures and activities. A previous study reported that some of the alkaloids isolated from *R. stricta*, such as akuammidine, rhazimanine, stemmadenine, strictanol, and tetrahydrosecaminediol, showed potential antimicrobial activity against different human pathogens such as *Pseudomonas aeruginosa, Escherichia coli, Staphylococcus aureus*, and *Candida albicans* [5].

*R. stricta* leaves, stems, roots, and legumes, as well as mixes of aerial parts, have yielded over 100 alkaloids that have been isolated, described, and named [6]. *R. stricta* has yielded the monomeric indole alkaloid 16-epi-Z-isositsirikine, which has antineoplastic action. Tetrahydrosecamine has antibacterial and anticancer properties [7]. Stemmadenine is an *R. stricta* antimicrobial alkaloid [8]. Aspidospermiose, aspidospermidose, leepacine, strictanine, strictibine, and strictanol are associated with species-specific transient ischaemic attack, and they have only been reported from *R. stricta* [3].

Plant fungal diseases complicate farmers’ plant output. Plant fungal infections cause devastating diseases all over the world [9]. *Fusarium* spp., for example, are well-known filamentous phytopathogens that diminishes crop quality, whereas *Blumeria* spp. lower crop amount. Infections can completely shut down plant immune responses when they coexist with other infections, such as *Fusarium graminearum* with other *Fusarium* species. Fungal infections generate a wide range of issues, with the potential to have a massive influence on plant output [10]. *Fusarium* is classified in the phylum *Ascomycota*, the subphylum *Pezizomycotina*, the class *Sordariomycetes*, the order *Hypocreales*, and the family *Nectriaceae*. *Fusarium* is a genus with about 1500 species. *Fusarium* spp. are filamentous fungi that are extensively distributed in the environment, and various strains cause illnesses in plants and animals, as well as producing mycotoxins [11]. *Fusarium solani* has been identified from soil and plant residues and linked to severe invasive infections in immunocompromised people. This species is present in soil and plant material; however, it is a pathogen that only affects industrially essential plants. Furthermore, they produce opportunistic infections in humans, which have a high fatality rate [12]. *Fusarium proliferatum* is found all over the world and has been connected to a variety of plant diseases. It may additionally induce infections in immunocompromised people and abscesses in plant-caused wounds [13]. All of these facts increased the need for more investigational studies about some natural fungicidal materials that might affect the mycelial growth of different *Fusarium* spp.

However, fungicides are frequently hazardous to humans (genotoxicity) and other untargeted creatures, contributing to ecological imbalances [10,14]. These compounds decay slowly and increase the population of the environment and water systems. As a result, different biological methods for controlling pathogenic fungi are needed [10].

Nanotechnology is defined as the creation, display, manipulation, and use of nanoparticles (NPs) [15]. Nanoparticles (NPs) have a single dimension ranging from 1 to 100 nm. NPs have distinguishing characteristics that set them apart from bulk materials [16]. Because of the stabilizing factors that allow NPs to readily interact with other biomolecules and increase their interactions with bacteria, biologically generated NPs have been shown to possess potential antimicrobial efficacy against different species [15]. The value of plant extracts is determined by their selection [17,18]. Prior research found that pH might affect the characteristics of the synthesized NPs. It was found that an acidic pH of 4.5 increased the NPs antibacterial action against *E. coli* but had less activity against *S. aureus* and *C. albicans* [19,20]. This emphasizes the significance of pH and its function in the display of the NP’s biological and medicinal effects.

The principles of AgNPs activity can be summarized in their capacity to bind to the cell surface and membrane, as well as cell penetration, producing oxidative damage by creating oxidative stress [21]. AgNPs can also affect microbial DNA by attaching to phosphate groups [17]. Furthermore, by interacting with proteins and enzymes, AgNPs can induce persistent cell damage by disrupting the electron transport chain, resulting in membrane permeability barrier disruption [22].

A previous study discovered that *R. stricta* root extract may be used to create AgNPs. AgNPs produced had an average size of 20 nm and a spherical form. The addition of *R. stricta* root extract caused the color of the AgNPs to shift from pale yellow to orange-brown. The results indicated that these AgNPs made using *R. stricta* root extract and xylitol exhibited potential antibacterial action since they were monodispersed, stable, and had minimal aggregation [20]. These studies showed that biologically produced NPs outperform chemically or physically created NPs. This shows that the potential of *R. stricta* extract and its fraction as a mechanism of biogenesis of NPs should be investigated further.

Therefore, the current study aimed to evaluate the antifungal properties of Aq-extract of *R. stricta* and its alkaline fraction against four *Fusarium* spp. Furthermore, the antifungal properties of biologically synthesized AgNPs from the crude Aq-and Alk-extracts of *R. stricta* were investigated as well.

## 2. Materials and Methods

### 2.1. Preparation of Plant Extracts

*R. stricta* leaves were taken from the desert near Um Muslaim (24°56’52” N, 45°42’37” E), 140 km from Riyadh Region, Saudi Arabia. The samples were recognized and validated at King Saud University’s College of Science, Riyadh, Saudi Arabia. Following that, the plant components (leaves) were cleaned with distilled water to eliminate dirt and debris. The samples were shade-dried before being processed into a course, fine powder using a milling machine. The finely powdered materials were then kept at room temperature in airtight plastic containers for extraction and subsequent analysis.

*R. stricta* powdered leaves (10 g) were steeped in 100 mL of distilled water (3 × 100 mL) and heated until ebullition occurred. The Aq-extract was mixed and filtered through muslin cloth before being filtered through Whatman filter paper No. 1 (Sigma-Aldrich, St. Louis, MO, USA) to remove water. The filtrates were vacuum distilled at decreased pressure at 50 °C using a Rotavapor^®^ R-300 (BÜCHI Labortechnik AG, Flawil, Switzerland). Then, the filtrate was concentrated and lyophilized to provide an Aq-extract containing about 40 g of dark brown residue.

The alkaline fraction of *R. stricta* was generated in the same manner as previously described [23,24]. The residue of *R. stricta* filtration was removed and filtered using Whatman filter paper at 80 °C for 30 min with 100 cc of 0.15 M NaOH. To remove the excess of NaOH, the filtrate was neutralized with HCl, condensed, and lyophilized to obtain the Alk-extract.

### 2.2. Preparation of AgNPs

In a round-bottom flask, 10 mL of *R. stricta* Aq-extract was mixed with 90 mL of the aqueous solution AgNO_3_ (1 mM) to make biogenic Aq-AgNPs (Aq-AgNPs). The flask had a magnetic stir bar connected to a cooling condenser. The reaction mixture was heated for an hour at 85–90 °C with steady magnetic stirring. Then, 5 mL of NaOH solution was added dropwise to the reaction mixture, and the color of the reaction mixture instantly changed from light yellow to dark brown. After cooling, the reaction mixture was centrifuged for 30 min at 9000 rpm. The resultant black residue was rinsed with deionized water and dried in an oven at 80 °C for 12 h [25].

Similarly, in a 250 mL round-bottom flask, 10 mL of the Alk-extract was treated with 90 mL of AgNO_3_ (1 mM) under continuous magnetic stirring for 1 h at 85–90 °C using a magnetic stir bar (VELP scientica Srl, Usmate, Italy). Then, 5 mL of NaOH solution was added dropwise to the reaction mixture to enhance the reaction velocity [26]. The color soon changed from pale yellow to brown. The reaction mixture was then allowed to cool before being centrifuged for 30 min at 9000 rpm and filtered. Finally, each reaction mixture’s result was rinsed multiple times with deionized water, and the precipitates generated were dried out separately in an oven for 12 h at 80 °C to yield the Alk-AgNPs

### 2.3. Characterization of Biogenic AgNPs

For the characterization of AgNPs, a UV-visible spectrophotometer, UV-2450 double-beam machine (Shimadzu, Tokyo, Japan) was employed. The UV absorption induced by the tested materials was measured at 200–900 nm, according to the manufacturer instructions.

Fourier transform infrared spectroscopy (FTIR) was used to identify the functional groups included in all preparations. At a range of 500–4000 cm^−1^, Nicolet™ iS50 FTIR spectrometer (Thermo Scientific, Waltham, MA, USA) was used to identify the absorbance values. The machine was equipped with an automated beam splitter exchanger, three detectors, and OMNIC Mercury TGA software version 9. Sample processing and preparation were according to the guidelines of the manufacturer. Identification of the functional groups depends on their percentage of transmittance, shape (broad or sharp), and strength (weak, medium, or strong) [23,25].

Dynamic light scattering (DLS) was used to determine the size (z-average) of the AgNPs in a colloidal suspension. According to the manufacturer’s instructions, a zeta sizer nanodevice (Malvern, Worcestershire, UK) was used to assess z-average (d. nm) of various biosynthesized AgNPs [27].

Transmission Electron Microscopy (TEM) analysis using JEOL JEM-1400 microscopy (JEOL, Peabody, MA, USA) was used to characterize the size, shape, and crystallinity of each biosynthesized AgNPs. Each test sample was placed in an 8-L container on a carbon-coated copper grid with a mesh size of 300. Images were obtained at a 200-kV acceleration voltage.

### 2.4. Fusarium Species

The Department of Plant Protection, College of Food and Agriculture Sciences, King Saud University, Riyadh, Saudi Arabia, provided and identified the *Fusarium* spp. *Fusarium solani, Fusarium incarnatum, Fusarium verticillioides,* and *Fusarium proliferatum* were the fungal species selected. These strains were all kept in Potato dextrose Agar (PDA), as described previously [28]. Until usage, the strains were either refrigerated at 4 °C or sub-cultured once a month.

### 2.5. Evaluation of the Fungistatic Activity

The poison food approach was used to assess the antifungal activity of different treatments in vitro [29]. The crude extracts and the biosynthesized AgNPs were diluted at different concentrations with distilled water and sterilized by passing through a 0.45 m bacterial filter. Following that, 1 mL of each concentration was diluted in 19 mL of the cooled molten PDA (45 °C) and mixed gently by rotation to ensure efficient dispersion of tested materials [30]. The final concentrations of extracts were 0%, 5%, 10%, and 20%, whereas for AgNPs, they were 0%, 25%, 50%, 75%, and 100% in PDA. A mycelial plug (6 mm), from the perimeter of a nine-day-old actively expanding colony, was inserted in the center of the PDA plate. Petri plates were incubated at 25 ± 2 °C after inoculation. In triplicate, all fungal strains were exposed to different concentrations of treatments (5, 10, and 20% of the Aq-extract, and 25, 50, 75, and 100 ppm of the pre-synthesized AgNPs). The control group received no treatment and only the media and test fungus. Fungicide (Previcur Energy, Bayer CropScience Ltd., Cambridge, UK) was employed as a control. When the control plate demonstrated complete plate development, the diameter of mycelial growth was measured. The growth area was calculated in cm^2^ by measuring the radius (r) of the culture plate and ZOI and calculating the area by the equation:

A = πr^2^

where A is the area calculated in cm^2^, r is the radius of the target area, and π is the mathematical constant equal to 3.14159.

The net growth area (GA) was calculated as follows:

GA = A_t_ − A_ZOI_

where A_t_ is the total culture area and A_ZOI_ is the area of zone of inhibition.

The percentage of the inhibition of mycelial growth (IMG) was computed as follows:

IMG % = (AC − AT)/AC × 100

where AC represents the colony diameter in the control plate and AT represents the colony diameter in the treated petri plates. All experiments were repeated three times for statistical reasons.

### 2.6. Scanning of the Fungal Growth Inhibition and Produced Germs by Light Microscopy

Small (6 mm) discs were aseptically extracted from mycelia areas on either the control (untreated) or treated plates. Disks were transferred to sterile slides without medium, stained with two drops of lactophenol dye (with a cover slip), and inspected under a light microscope at 10× and 40× magnification.

### 2.7. Statistical Analysis

For statistical purposes, the experimental experiments were carried out in duplicates. The statistical analysis was carried out using IBM’s Statistical Package for the Social Sciences (SPSS) version 22 (Armonk, NY, USA). One-way ANOVA analysis accompanied by the method of the least significant difference (LSD) for the post-hoc multiple comparisons test was used to compare the means between different treatments groups. If the *p*-values were less than 0.05, the results were considered significant.

## 3. Results

The extraction of *R. stricta* leaves resulted in a good yield of aqueous (dark green) and aqueous alkaline (dark brown) fractions of 40 g and 30.1 g, respectively (Supplementary Figure S1).

In the current investigation, the biosynthesis of AgNPs was driven by the reduction of Ag^+^ to Ag^0^, which was visible as a shift in the color of the reaction mixture from light yellow to dark brown. For the characterization and identification of pre-synthesized AgNPs, several spectroscopic (FTIR, UV-vis and DLS) and microscopic (TEM) investigations were conducted.

**Figure 1:**
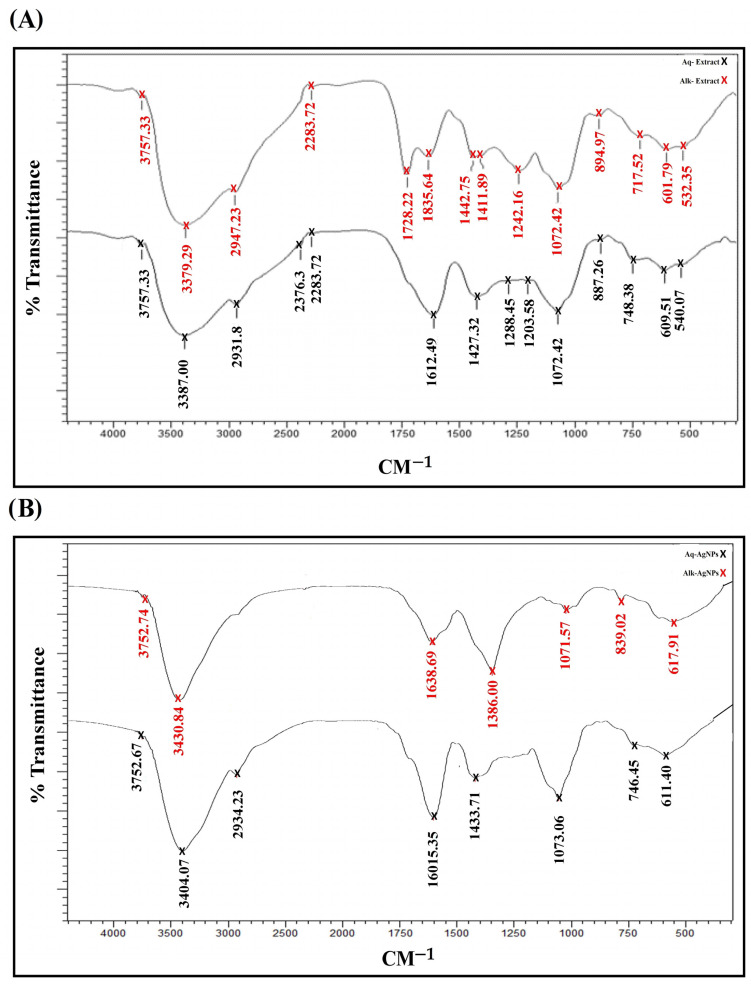
FTIR spectra of *R. stricta* preparations. (**A**) *R. stricta* Aq-Extract and Alk-fraction, (**B**) *R. stricta* Aq-AgNPs and Alk-AgNPs.

The FTIR analysis (Figure 1) showed that all preparations were rich in alcohol (O-H stretching), amines (C-N stretching), and halo compounds (C-Br or C-I stretching). Except the Alk-AgNPs, the other preparations showed the existence of alkanes (C-H stretching), amines (N-H bending), and carboxylic acids (O-H bending). Unlike AgNPs, both of the prepared extracts had functional groups of Isocyanate (N=C=O stretching) and Alkenes (C=C bending). Alk-extract and AgNPs were distinguished from other preparations and from each other by two functional groups at 1728.22 cm^−1^ for Aldehyde (C=O stretching) and at 1638.69 cm^−1^ for Alkene (C=C stretching). The Aq-extract of *R. stricta* showed a weak sharp peak at 2376.3 cm^−1^, which indicated the existence of secondary amine halide salts (NH^2+^ stretches). Readings above 3700 cm^−1^ indicated noise peaks which were neglected (Figure 1, Table 1).

**Figure 2:**
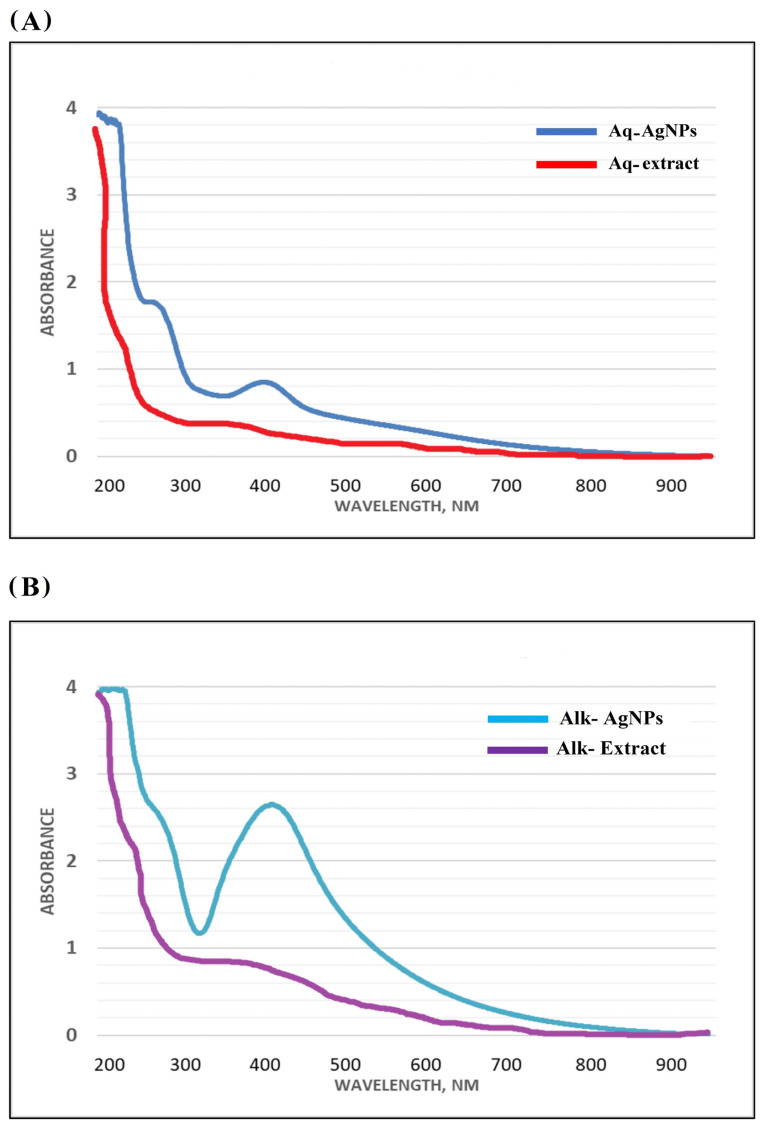
UV-vis spectra of AgNPs prepared from *R. stricta* leaves extract compared to the crude extracts. (**A**) Aq-extract and Aq-AgNPs, (**B**) Alk-extract and Alk-AgNPs.

The UV-visible spectra of AgNPs were measured between 200 and 900 nm. The UV-vis spectra of AgNPs produced from Aq-extract and Alk-fraction revealed two large peaks at 405 and 415 nm, respectively (Figure 2). The peak of Alk-AgNPs is greater than that of Aq-AgNPs, as shown in Figure 2B, which might be related to higher energy consumption by the NPs due to the high amount of bonding. It was noticed that there is a shoulder peak at 250–270 nm of the AgNPs spectra, which might be due to the vibrational and vibro-rotational transitions of AgNPs [31]. The UV spectra of crude extracts did not produce any significant peaks.

**Figure 3:**
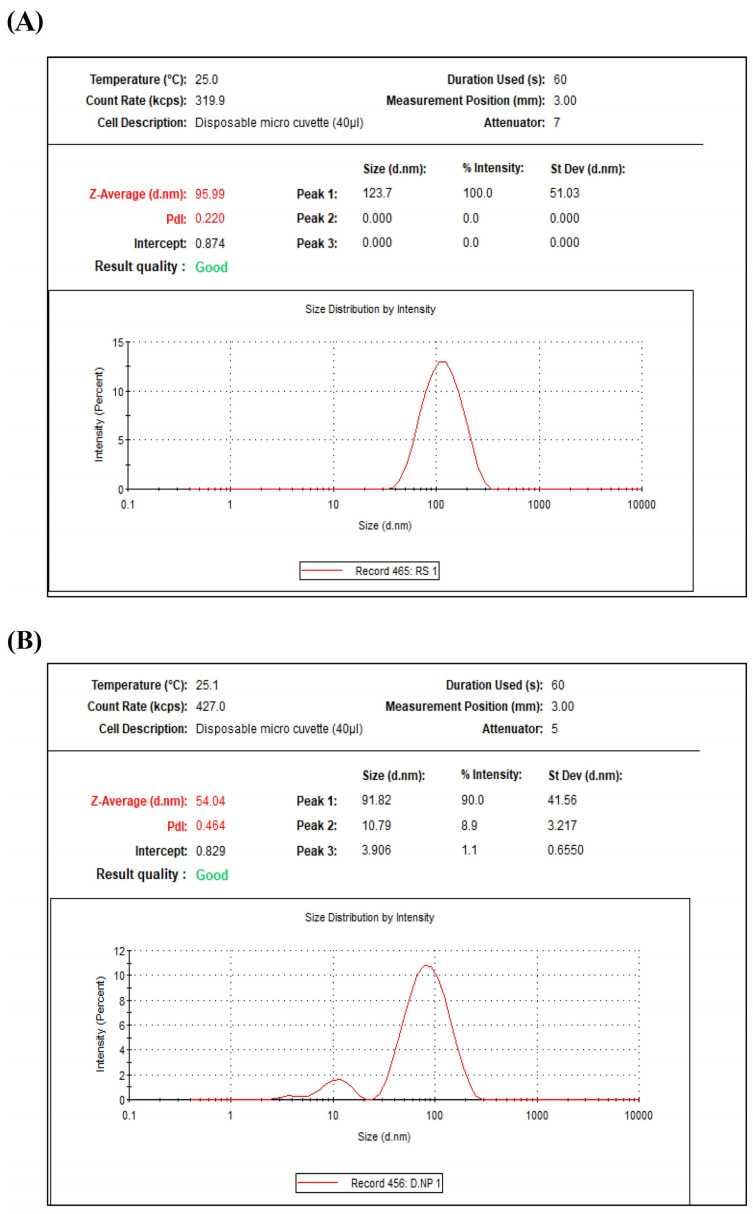
Z-average size of biosynthesized AgNPs of *R. stricta* leaves extract. The particle size distribution was measured by zeta sizer nanodevice. (**A**) Aq-AgNPs, (**B**) Alk-AgNPs.

Right after synthesis, the average particle size, diameter, and polydispersity indices (PDI) of all pre-synthesized AgNPs were assessed. Aq-AgNPs and Alk-AgNPs had average particle sizes (z-average) of 95.9 nm (PDI value 0.220, intercept 0.874) and 54.04 nm (PDI value 0.464, intercept 0.829), respectively (Figure 3). This demonstrates a variation in particle size between the two preparations, which might be attributed to pH differences.

**Figure 4:**
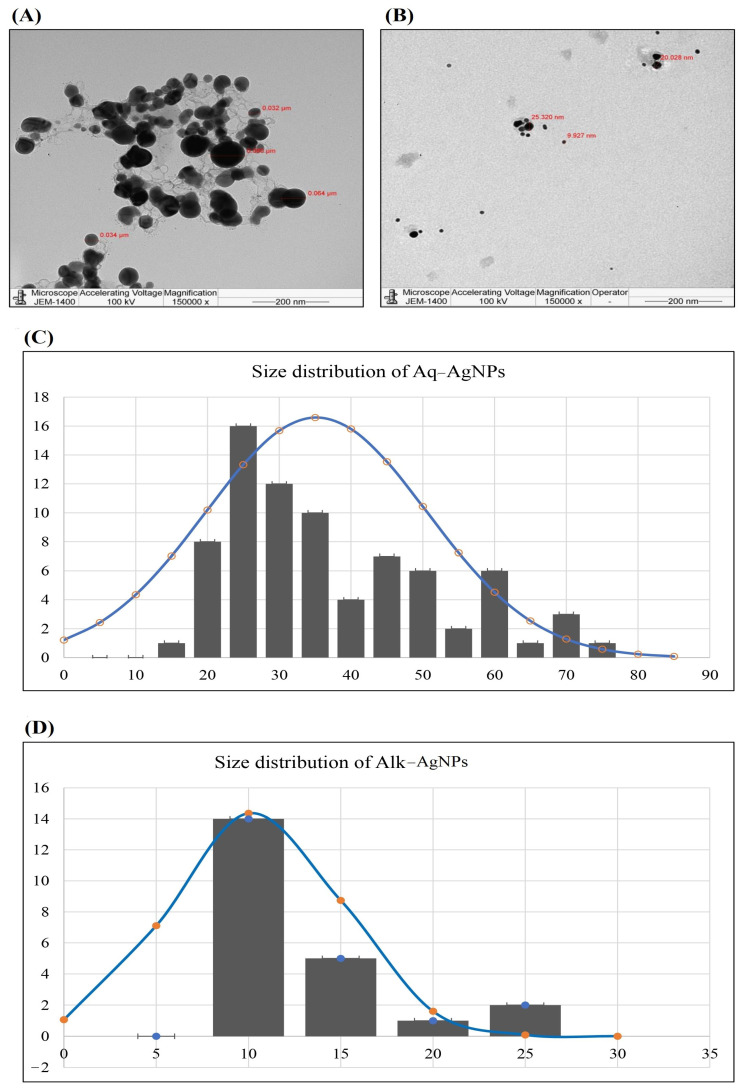
TEM microphotographs and size distribution histograms of the biogenic AgNPs of *R. stricta* extracts. (**A**) Aq-AgNPs, (**B**) Alk-AgNPs, (**C**) size distribution histogram of Aq-AgNPs, (**D**) size distribution histogram of Alk-AgNPs.

The size of biosynthesized AgNPs was evaluated using TEM imaging to validate the zetasizer results. The picture formed from the transmitted electrons is directly observed by TEM. It describes the structural and chemical behavior of NPs at high electron density and resolution. Various scientists have used TEM to classify and monitor green produced AgNPs. The surface shape and size of pre-synthesized AgNPs from *R. stricta* leaf extracts and fractions were determined using TEM. The results showed that both of the biosynthesized Aq-AgNPs were well disseminated, aggregated, and that they all seemed to be spherical, while the Alk-AgNPs were spherical with no aggregation. Aq-AgNPs and Alk-AgNPs had average diameters ranging from 21 to 90 nm and 7.249 to 25.32 nm, respectively (Figure 4). As noticed, the sizes of Alk-AgNPs were smaller than that of Aq-AgNPs, which is in accordance with the DLS analysis.

The antifungal activity of *R. stricta* extracts and biogenic AgNPs was evaluated separately in vitro on PDA medium against selected saprophytic fungi, *F. solani, F. incarnatum, F. verticillioides,* and *F. proliferatum*, for antifungal activity and the possibility of improving their applications by measuring mycelial growth diameter.

**Figure 5:**
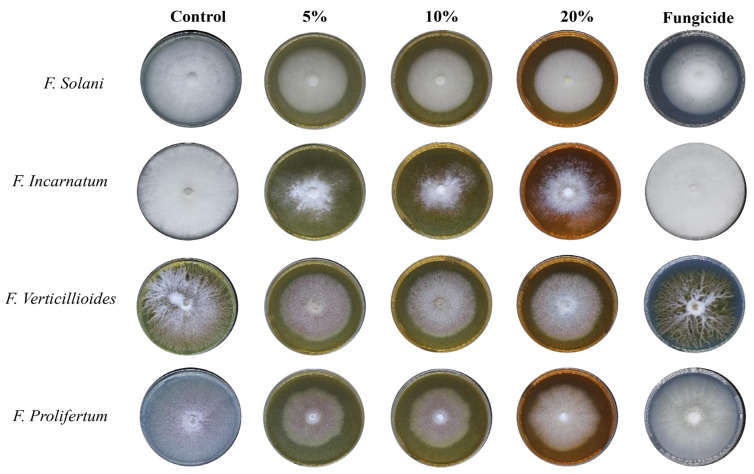
The antifungal activity of *R. stricta* Aq-extract.

The Aq-extract of *R. stricta* showed mild inhibition at different concentrations (5, 10, and 20 mg/mL) against all selected fungal species (*F. solani, F. incarnatum, F. verticillioides,* and *F. proliferatum*) with different percentages of inhibition. At a concentration of 20%, the Aq-extract of *R. stricta* demonstrated considerable inhibition (Table 1 and Figure 5), with notable (*p* < 0.001) growth suppression against *F. incarnatum, F. solani, F. proliferatum*, and *F. verticillioides*, with 19.8%, 28.1%, 37.5%, and 27.1%, respectively (Table 2 and Figure 5).

**Figure 6:**
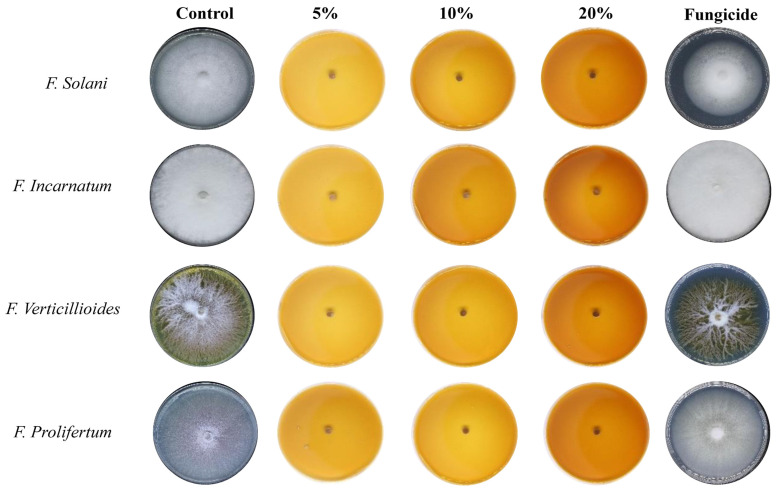
The antifungal activity of *R. stricta* Alk-extract.

For the species treated with different doses of the Alk-extract of *R. stricta,* the results showed stronger effects. The 10% and 20% concentrations caused complete inhibition of the mycelial growth, compared to the positive and negative controls (Table 2, Figure 6). Notably, the antifungal effects of the lower concentration of Previcur Energy were insignificant, which might be explained by the fact that these species showed robust resistance.

**Figure 7:**
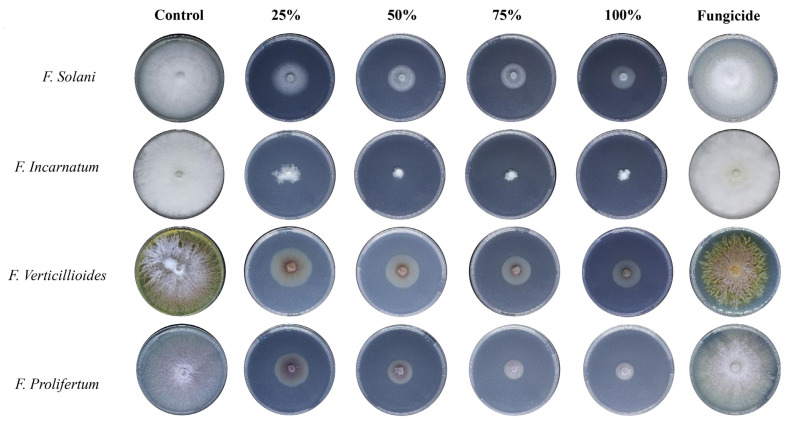
The antifungal activity of *R. stricta* Aq-AgNPs.

However, the observed growth area of mycelial development decreased as the concentration of biosynthesized AgNPs (25, 50, 75, and 100%) increased against selected fungal species (*F. solani, F. incarnatum, F. verticillioides*, and *F. proliferatum*) (Table 3 and Figure 7). The results demonstrated considerable suppression of mycelium growth, as evidenced by a decrease in growth diameter at 100% concentration of Aq-AgNPs. Biogenic Aq-AgNPs inhibited *F. incarnatum, F. solani, F. proliferatum*, and *F. verticillioides* growth by 87.5%, 72.3%, 75%, and 62.5%, respectively (*p* < 0.05). The results showed that *F. incarnatum* was the most affected species (87.5%), whereas *F. verticillioides* grew the least (62.5%).

**Figure 8:**
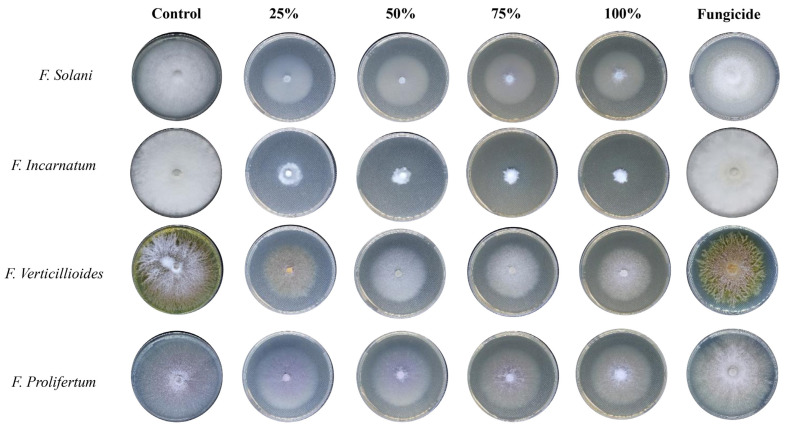
The antifungal activity of *R. stricta* Alk-AgNPs.

The results exhibited moderate inhibition of mycelium growth at 100% of the Alk-AgNPs, with 87.8%, 37.5%, 37.5%, and 29.1% growth inhibition against *F. incarnatum, F. solani, F. verticillioides,* and *F. proliferatum*, respectively. It is evident that *F. incarnatum* was the most affected species (87.8%), while *F. proliferatum* was the least affected (29.1%) (Table 3 and Figure 8).

It was observed that the control (without treatment) growth of *F. solani* exhibited a cottony, white, and abundant mycelium. Additionally, treatment with fungicide (Previcur Energy) resulted in lower-density growth with no morphological changes. After treatment with Aq-extract, the observed growth was cottony white with high density, while the biogenic AgNPs resulted in scarce vertical, white mycelium growth. Finally, the Alk-fraction exhibited complete inhibition; however, the biogenic AgNPs showed moderate inhibition with horizontal growth and low density. It appears that the Alk-fraction was the most effective treatment against *F. solani*, followed by the Aq-extract.

The control of *F. incarnatum* showed abundant cottony white mycelium growth. Additionally, the treatment with fungicide (Previcur Energy) did not cause any morphological changes. Further, the Aq-extract resulted in weak growth with low density, while treatment with all biogenic AgNPs resulted in scarce vertical mycelium growth.

In *F. verticillioides*, the mycelium growth in the control appears cottony white with yellow pigmentation in the media. Further, a cottony growth with low density was observed after treatment with Aq-extract, while the biogenic AgNPs resulted in a flat growth with a reddish-brown center. Finally, the Alk-fraction caused total growth inhibition; however, AgNPs resulted in horizontal mycelium growth with low density. The fungus was most sensitive to the Alk- fraction, followed by the Aq-extract.

The control of *F. proliferatum* exhibited abundant growth with purple pigmentation, while the treatment with fungicide (Previcur Energy) showed an absence of purple pigment. The treatment with Aq-extract showed horizontal growth with low density while using AgNPs resulted in scarce vertical growth. Finally, the treatment with alkaline acid AgNPs showed low-density vertical growth with purple pigmentation. It appears that the Alk-fraction was the most effective treatment against *F. proliferatum*, followed by the Aq-extract.

The microscopic examination of fungal samples from the control (untreated) or treated plates was performed by light microscopy. The light observation of all tested fungal strains exhibited differences from the natural form and size of hyphae and conidiophores.

**Figure 9:**
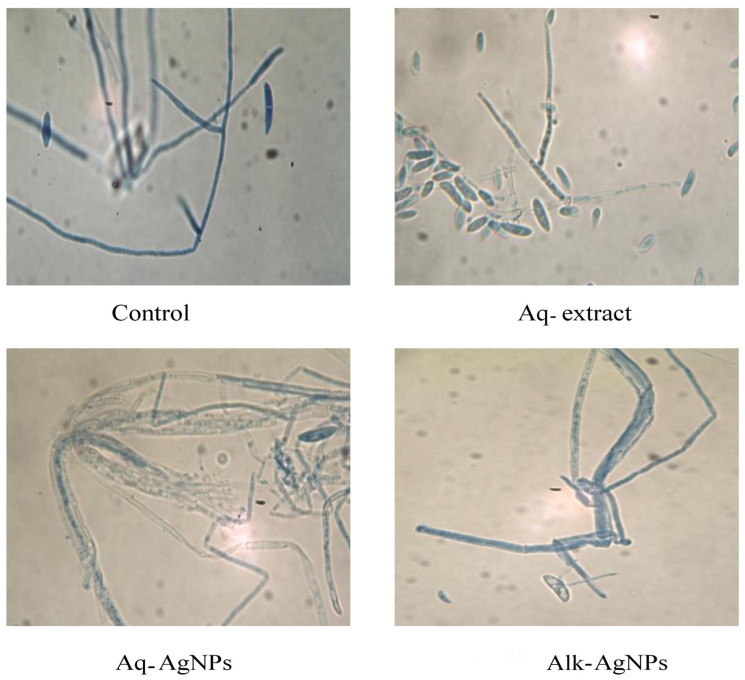
Light microscopic images displayed the morphological changes of *F. solani* in response to treatment with *R. stricta* Aq-extract and biosynthesized AgNPs at 40× magnification.

The images of the untreated *F. solani* species showed the macroconidia were oval, elongated or obovoid, septated, and reniform, with a remarkable truncated base. The filaments appeared septated and hyaline and the hyphae were narrow and branched (Figure 9). Treatment with Aq-extract induced limited effects that appeared in short hyphae and less elongated conidiophores. Both AgNPs induced the thickening and aggregation of hyphae, which were less branched, and the macroconidia had a more condensed shape and lighter color. It was obvious that Aq-AgNPs had a stronger effect than Alk-AgNPs, where the hyphae looked ruptured with a fewer number of conidia.

**Figure 10:**
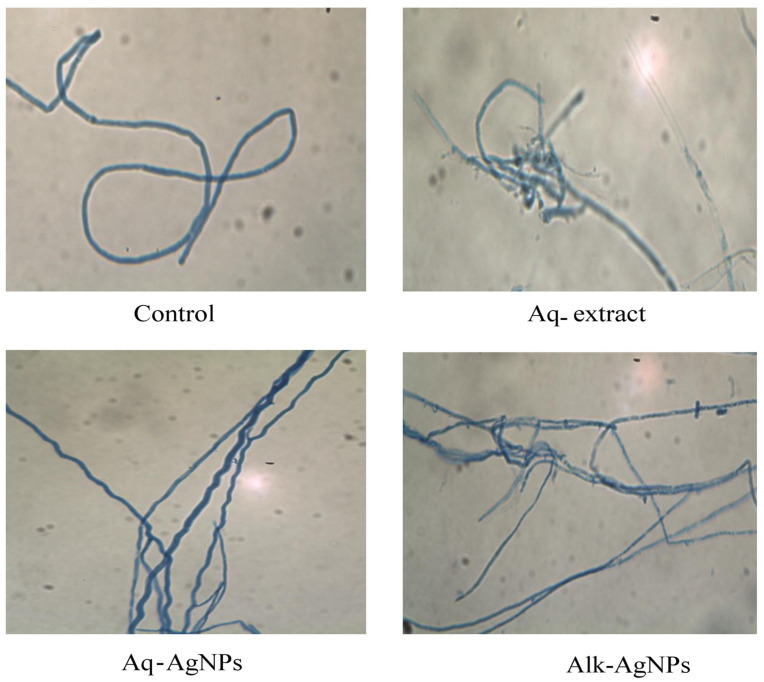
Light microscopic images displayed the morphological changes of *F. incarnatum* in response to treatment with *R. stricta* Aq-extract and biosynthesized AgNPs at 40× magnification.

The control image of *F. incarnatum* revealed abundant structures of filaments and spores. The intercalary chlamydospores were not identified by the light microscope, while the Ariel mycelium was simple, unbranched, smooth and thin walled (Figure 10). It was noticed that the hyphae treated with Aq-AgNPs appeared in zigzag shape, where the hyphae treated with Alk-AgNPs were thinner and more condensed that other dishes.

**Figure 11:**
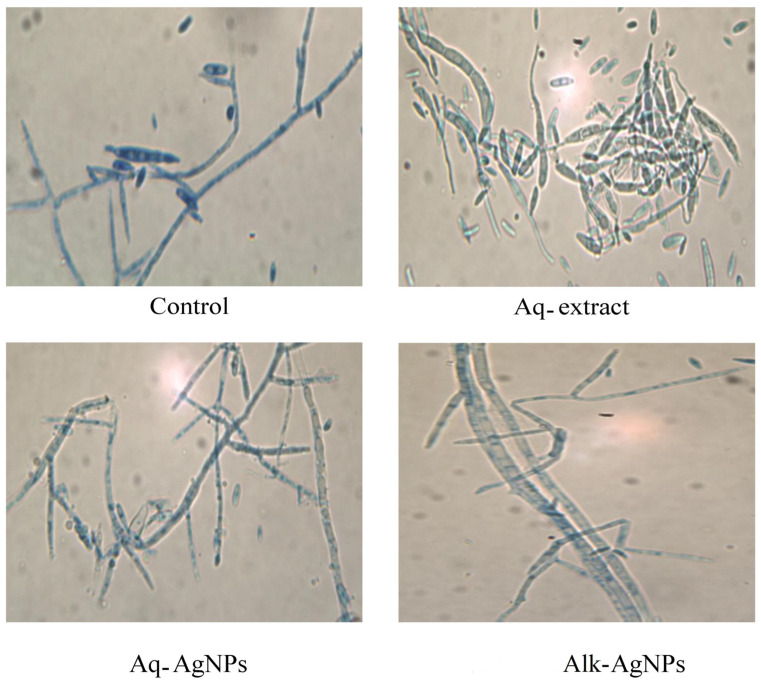
Light microscopic images displayed the morphological changes of *F. verticillioides* in response to treatment with *R. stricta* Aq-extract and biosynthesized AgNPs at 40× magnification.

The control image for *F. verticillioides* showed clusters of microconidia generated from monophialides. The microconidia were club-shaped with a flattened base, and the monophialides were V-shaped with few macroconidia. The light microscope did not reveal any chlamydospores. The filamentous hyphae were dark, thin, and smooth (Figure 11). Treatment with Aq-extract of *R. stricta* causes the swelling of the hyphae with aggregation of more macroconidia than in the control. Treatment with both of the biosynthesized AgNPs induced more swelling of the hyphae and the disappearance of conidia, particularly with the Alk-AgNPs.

**Figure 12:**
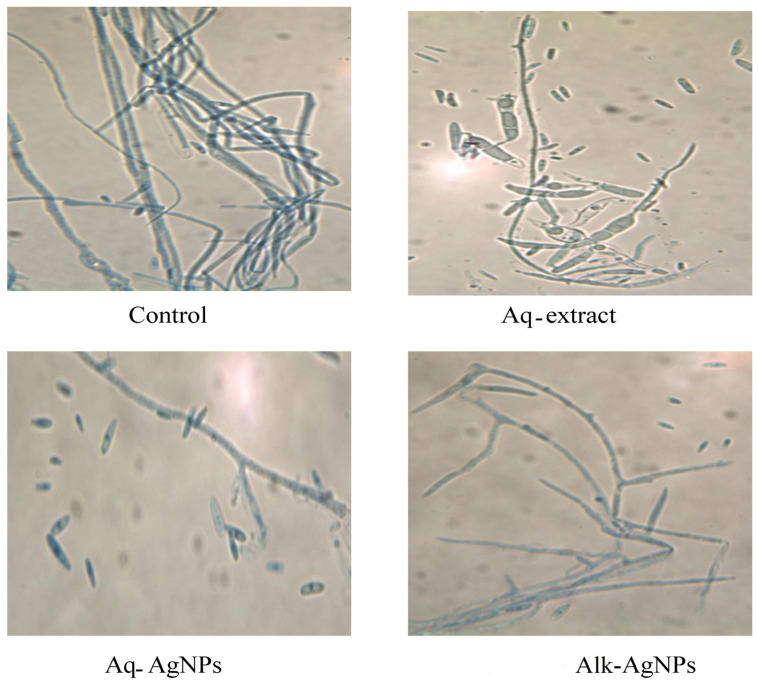
Light microscopic images displayed the morphological changes of *F. proliferatum* in response to treatment with *R. stricta* Aq-extract and biosynthesized AgNPs at 40× magnification.

Finally, the microscopic images of *F. proliferatum* showed remarkable morphological changes upon treatment with AgNPs and an Aq-extract of *R. stricta*, compared to the control. The macroconidia were very rare and appeared slender, foot-shaped, and slightly curved. Chlamydospores were not formed (Figure 12). Treatment with the Aq-extract of *R. stricta* or the biosynthesized AgNPs caused the swelling of spores and decreased the branching of hyphae, which appeared pale and ruptured. *F. proliferatum* lacked spores after treatment with the Aq-extract and biogenic AgNPs compared to control.

## 4. Discussion

*R. stricta* is a member of the *Apocynaceae* family, which contains alkaloids, cardenolides, triterpenoids, phenols, and iridoids. These components determine their biological significance or toxicity [4]. Previous research has also indicated a wide variety of pharmacological effects, including anti-inflammatory, cardioprotective, hepatoprotective, hypoglycemic, and neuroprotective qualities [6,32].

NPs have sparked interest due to their distinct properties that set them apart from bulk materials [33]. The effect of size and other special physical qualities (i.e., form and structure) on NPs results in unique properties [16]. Furthermore, physiologically generated NPs have unique features when compared to physiochemically manufactured NPs [34].

The current work used *R. stricta* Aq-extract and the alkaline fraction to manufacture different AgNPs. A detailed characterization was required for assessing and comprehending the biological activity of biogenic AgNPs. These distinguishing characteristics include AgNP size, shape, size distribution, and aggregation [35]. Biosynthesized AgNPs were evaluated using FTIR, UV-vis, DLS spectroscopic analyses, and TEM microscopic imaging.

All of the produced extracts and NPs were analyzed using FTIR to identify the compounds that operate as stabilizing and coating agents, as well as to detect silver ion reduction. The results showed that all preparations had functional groups of alcohol, amines, and halo compounds. The *R. stricta* Aq-extract and its alkaline fraction had Isocyanate and Alkene groups. In accordance with our results, a previous study showed that the aqueous and ethanolic extracts of *R. stricta* revealed they were rich in carboxyl, carbonyl, alcohol, aldehydes, and phenols, which might act as reducing agents in the synthesis of AgNPs [36].

The UV-visible spectrum of AgNPs was studied in the current work, and broad peaks were found in the UV-vis spectra of AgNPs generated from Aq-extract (405 nm) and Alk-fraction (415 nm). The observed enhanced shift of the UV peak might be attributed to NPs agglomeration caused by AgNPs assembly and the presence of several secondary metabolites that interact with the silver nitrate in the reaction solution [37]. Previous research has shown that AgNPs biosynthesized from the Aq-extract of *R. stricta* displayed wide peaks at 405–420 nm [37,38,39,40].

In the current investigation, the DLS approach was employed to estimate the diameter of synthesized AgNPs dispersed in liquid. As it can determine the size of the AgNPs colloidal solution, this technique has been frequently used to evaluate AgNPs generated utilizing phytochemical substances [27]. The idea of this technique is that it disperses the particles in the colloidal solution and scatters the light, resulting in an image of the particle and determining the distribution size in the 3–10 m range [40]. The average particle size, diameter, and polydispersity of all pre-synthesized AgNPs were measured immediately after synthesis. The average particle size (z-average) of Aq-AgNPs and Alk-AgNPs were 95.9 nm and 54.04 nm, respectively, with PI values of 0.22 and 0.464, according to the data. The z-average of Alk-AgNPs is smaller than that of Aq-AgNPs, which could be attributed to the increased Brownian motion of silver ions and, therefore, the creation of bigger particles of Aq-AgNPs [41]. In addition, the use of NaOH for pH adjustment might contribute to the conversion of AgNO_3_ into brown Ag_2_O precipitate and further producing smaller NPs [42].

Finally, at a high electron density and resolution, TEM was employed to gain insight into the structural and chemical behavior of biosynthesized AgNPs. TEM has been used by several scientists for the classification and monitoring of green-produced AgNPs. As nanoparticles have different physicochemical properties depending on their form and size, TEM is regarded as an important tool for assessing the size, shape, and dispersion of NPs [43]. TEM revealed that the biosynthesized Aq-AgNPs were spherical and distributed efficiently and aggregated, while Alk-AgNPs had no agglomeration. The average sizes of Aq-AgNPs were 21–90 nm and 7.249–25.32 nm for Alk-AgNPs. In agreement with these investigations, previous studies reported that AgNPs created from the methanolic and Aq-extracts of *R. stricta* root extract had spherical shapes with average sizes of 20–35 nm [44].

The most prevalent cause of food rotting has been identified as a variety of phytopathogenic fungi. Furthermore, certain fungi can create and release mycotoxins, which are toxic to humans and animals. To prevent such illnesses, chemical fungicides and pesticides have been utilized; nevertheless, these treatments have had a significant influence on the environment and, as a result, human health [45]. Plant extracts and plant-based AgNPs were used in this work to assess their effectiveness against four phytopathogenic *Fusarium* species. The Aq-extract of *R. stricta* leaves inhibited all of the fungal species examined. Growth observation of all examined fungi revealed a considerable shift in terms of growth density, color change, and perceived growth weakening. Furthermore, all pre-synthesized AgNPs from *R. stricta* showed outstanding efficacy against the tested fungi, with Aq-AgNPs more effective than Alk-AgNPs. This might be due to the participation of specific molecules in the green synthesis process that contribute to reducing silver ions, which impacts the antifungal activity of biogenic AgNPs [44]. Furthermore, the light microscope was utilized to study the damage induced by *R. stricta* leaves extract and all biogenic AgNPs. The detected fungal hyphae membrane showed damage to its integrity, making the cell susceptible and prone to death.

In agreement with our findings, a previous study showed that the Aq-extract of *R. stricta* affected the mycelial growth of *F. solani* with a zone of inhibition (ZOI) of 16 ± 0.3 mm, where the biosynthesized AgNPs induced a ZOI of 26 ± 0.5 mm [36]. Another study showed that the methanolic extract of *R. stricta* affected the mycelial growth of *F. solani* with a ZOI of 18 mm [45,46].

Despite fewer studies about the antifungal activities of *R. stricta* against *Fusarium* species, it has been shown to have significant antimicrobial effects against different bacterial, fungal, and candida species. For example, the methanolic and ethanolic extracts of *R. stricta* leaves showed significant antibacterial activity against *Klebsiella pneumoniae* [47]. Furthermore, the aqueous alkaloid and non-alkaloid extracts of *R. stricta* leaves reduced the growth of *S. aureus* [48,49] and *E. coli* [48]. The AgNPs biosynthesized from the methanolic extract of the *R. stricta* root showed an inhibitory effect against *B. subtilis* and *E. coli* [19]. Different strains of *S. aureus* and *Listeria monocytogenes* were sensitive to the treatment with 5–10 mg/mL of the essential oil of *R. stricta,* in the study conducted in Zabol, Iran [50]. Finally, a previous study from King Saud University, Riyadh, Saudi Arabia, reported that the methanolic extract of *R. stricta* had an inhibitory effect on the growth of *E. coli* (8.0 ± 1.0 mm), *S. aureus* (15.3 ± 0.5 mm), and *C. albicans* (19.3 ± 0.58 mm) [51]. These findings reveal that the Aq-extract and biologically generated NPs have unique antimicrobial properties, indicating that *R. stricta* extract and its fraction should be investigated further as a way of biosynthesis of NPs.

## 5. Conclusions

The findings suggested the antifungal activity of tested *R. stricta* Aq-extract and alkaline fraction against filamentous fungi, such as *Fusarium* species. The pre-synthesized Aq-AgNPs showed high activity compared to the Alk-AgNPs. The results showed that alkaline fraction was the most effective treatment among others. As a final conclusion, *F. verticillioides* was the most sensitive species to the different treatments of *R. stricta,* while *F. proliferatum* was the most resistance. The study also revealed that the fungicide Previcur Energy was less effective than all *R. stricta* preparations, which is the key result of the current study. These results confirm that *R. stricta*, which had different medical uses in folk medicine, had significant fungicidal effects against phytopathogenic *Fusarium* species. Further experimental research is needed about using plant extracts as an alternative to harmful chemicals, in addition to a deeper investigation into the ultra-cellular and molecular damage caused by *R. stricta* extract, to describe its possible antimicrobial mechanisms. In addition, further studies might be required to investigate the antifungal activities of other extracts or AgNPs of *R. stricta* and against other fungi including other *Fusarium* species.

## Figures and Tables

**Table 1:** FTIR spectra of the functional groups obtained from screening of prepared *R. stricta* extracts and biosynthesized AgNPs.

Compound Class	Functional Group	Aq-Extract	Alk-Extract	Aq-AgNPs	Alk-AgNPs
Alcohol	-hydroxyl (O-H stretching)	3757.33; 3387.00	3757.33; 3379.29	3752.67; 3404.07	3752.74; 3430.84
Alkane	-alkyl (C-H stretching)	2931.8	2947.23	2943.23	
Secondary amine halide salts	NH^2+^ stretches	2376.3			
Isocyanate	N=C=O stretching	2283.72	2283.72		
Aldehyde	C=O stretching		1728.22		
Alkene	-alkenyl (C=C stretching)				1638.69
Amine	-amino (N-H bending)	1612.49	1635.64	1601.15	
Carboxylic acid	Carboxyl (O-H bending)	1427.32	1442.75, 1411.89	1433.71	1386
Nitrile	Cyano (C-N stretching)	1288.45, 1203.58, 1072.42	1242.16, 1072.42	1073.06	1071.57
Alkene	alkenyl (C=C bending)	887.26	894.97		
Halo compounds	C-Br or C-I stretching	748.38, 609.51, 540.07	717.52, 601.79, 532.35	746.45, 611.4	839.02, 617.91

**Table 2:** Assessment of in vitro antifungal activity of *R. stricta* crude extracts.

Concentration	0%		5%		10%		20%		Previcur Energy (Fungicide)	
	GA *	IMG%	GA *	IMG%	GA *	IMG%	GA *	IMG%	GA *	IMG%
*R. stricta* Aq-extract										
*F. solani*	50.27 ± 0.0	0.0	47.12 ± 0.0	25.0	46.87± 0.05	26.0	46.29 ± 0.0	28.1	48.3 ± 0.1	19.8
*p-value #*	1		0.66		0.63		0.57		<0.001 #	
*F. verticillioides*	50.27 ± 0.0	0.0	47.12 ± 0.0	25.0	47.12 ± 0.07	25	46.57 ± 0.0	27.1	49.35 ± 0.06	13.5
*p-value #*	1		0.03#		0.66		0.60		<0.001 #	
*F. proliferatum*	50.27 ± 0.0	0.0	47.12 ± 0.07	25	43.57 ± 0.0	37.536.5	43.2 ± 0.0	37.5	49.48 ± 0.0	12.5
*p-value #*	1		0.66		0.34		0.32		<0.001 #	
*F. incarnatum*	50.27 ± 0.0	0.0	49.19 ± 0.08	14.6	48.3 ± 0.07	19.8	48.3 ± 0.06	19.8	50.27 ± 0.0	0.0
*p-value #*	1		0.88		0.78		0.78		1	
*R. stricta* Alk-extract										
*F. solani*	50.27 ± 0.0	0.0	11.78 ± 0.0	87.5	0.0 ± 0.0	100	0.0 ± 0.0	100	48.3 ± 0.1	19.8
*p-value #*	1		<0.001 #		<0.001 #		<0.001 #		<0.001 #	
*F. verticillioides*	50.27 ± 0.0	0.0	11.78 ± 0.0	87.5	0.0 ± 0.0	100	0.0 ± 0.0	100	49.35 ± 0.06	13.5
*p-value #*	1		<0.001 #		<0.001 #		<0.001 #		<0.001 #	
*F. proliferatum*	50.27 ± 0.0	0.0	11.78 ± 0.0	75.587.5	0.0 ± 0.0	100	0.0 ± 0.0	100	49.48 ± 0.0	12.5
*p-value #*	1		<0.001 #		<0.001 #		<0.001 #		<0.001 #	
*F. incarnatum*	50.27 ± 0.0	0.0	11.78 ± 0.0	87.5	0.0 ± 0.0	100	0.0 ± 0.0	100	50.27 ± 0.0	0.0
*p-value #*	1		0.561		0.561		<0.001 #		1	

* GA is the growth area calculated in CM^2^ and expressed as mean ± standard deviation. # One-way ANOVA tests and post-hoc comparisons (LSD test) were used to compare the growth rates of treated organisms against the untreated; it was considered significant at *p* < 0.05. IMG%: Percentage of mycelial growth inhibition; *F.*: *Fusarium.*

**Table 3:** Assessment of in vitro antifungal activities of AgNPs biosynthesized from *R. stricta.*

Concentration	0%		25%		50%		75%		100%		Previcur Energy (Fungicide)	
	Growth *	IMG%	Growth *	IMG%	Growth *	IMG%	Growth *	IMG%	Growth *	IMG%	Growth *	IMG%
*R. stricta* Aq-AgNPs												
*F. solani*	50.27 ± 0.0	0.0	34.71 ± 0.0	55.6	30.63 ± 0.0	62.5	26.51. ± 0.0	68.75	24.03 ± 0.0	72.3	48.3 ± 0.1	19.8
*p-value #*	1		<0.001 #		<0.001 #		<0.001 #		<0.001 #		<0.001 #	
*F. verticillioides*	50.27 ± 0.0	0.0	37.7 ± 0.0	50.0	32.54 ± 0.0	59.4	30.63 ± 0.0	62.5	30.63 ± 0	62.5	49.35 ± 0.06	13.5
*p-value #*	1		<0.001 #		<0.001 #		<0.001 #		<0.001 #		<0.001 #	
*F. proliferatum*	50.27 ± 0.0	0.0	33.28 ± 0.07	58.1	26.51 ± 0.0	68.8	21.99 ± 0.0	75.0	21.99 ± 0.0	75.0	49.48 ± 0.0	12.5
*p-value #*	1		<0.001 #		<0.001 #		<0.001 #		<0.001 #		<0.001 #	
*F. incarnatum*	50.27 ± 0.0	0.0	21.99 ± 0.0	75.0	11.78 ± 0.0	87.5	11.78 ± 0.0	87.5	10.011.78 ± 0.0	87.5	50.27 ± 0.0	0.0
*p-value #*	1		<0.001 #		<0.001 #		<0.001 #		<0.001 #		1	
*R. stricta* Alk-AgNPs												
*F. solani*	50.27 ± 0.0	0.0	43.2 ± 0.0	37.5	43.2 ± 0.0	37.5	43.2 ± 0.0	37.5	43.2 ± 0.0	37.5	48.3 ± 0.1	19.8
*p-value #*	1		<0.001 #		<0.001 #		<0.001 #		<0.001 #		<0.001 #	
*F. verticillioides*	50.27 ± 0.0	0.0	43.2 ± 0.0	37.5	43.2 ± 0.0	37.5	43.2 ± 0.0	37.5	43.2 ± 0.0	37.5	49.35 ± 0.06	13.5
*p-value #*	1		<0.001 #		<0.001 #		<0.001 #		<0.001 #		<0.001 #	
*F. proliferatum*	50.27 ± 0.0	0.0	47.12 ± 0.14	25	46.87 ± 0.0	26	46.57 ± 0.07	26.027.1	46 ± 0.07	29.1	49.48 ± 0.0	12.5
*p-value #*	1		<0.001 #		<0.001 #		<0.001 #		<0.001 #		<0.001 #	
*F. incarnatum*	50.27 ± 0.0	0.0	22.74 ± 0.13	74.0	17.89 ± 1.40.07	80.3	14.48 ± 2.50.13	84.4	12.511.78 ± 0.13	84.487.5	50.27 ± 0.0	0
*p-value #*	1		<0.001 #		<0.001 #		<0.001 #		<0.001 #		1	

* GA is the growth area calculated in CM^2^. Growth is and expressed as mean ± standard deviation. # One-way ANOVA tests and post-hoc comparisons (LSD test) were used to compare the growth rates of treated organisms against the untreated; it was considered significant at *p* < 0.05. IMG%: Percentage of mycelial growth inhibition; *F.*: *Fusarium.*

## Data Availability

All the data presented in this study are available in the article.

## Supplementary Materials

The following supporting information can be downloaded at: https://www.mdpi.com/article/10.3390/nano14010088/s1, Figure S1: Color change of the AgNPs biosynthesis process by *R. stricta* Aq-extract and its alkaline fraction.
