# Supplementary material for: The Antifungal Activities of Silver Nano-Aggregates Biosynthesized from the Aqueous Extract and the Alkaline Aqueous Fraction of Rhazya stricta against Some Fusarium Species
Source: Nanomaterials (Basel). 2023 Dec 28;14(1):88. doi: 10.3390/nano14010088 (PMC10780319; doi:10.3390/nano14010088)
Supplement: Supplementary file 1 [file nanomaterials-14-00088-s001.zip › nanomaterials-2758600-SI.pdf]

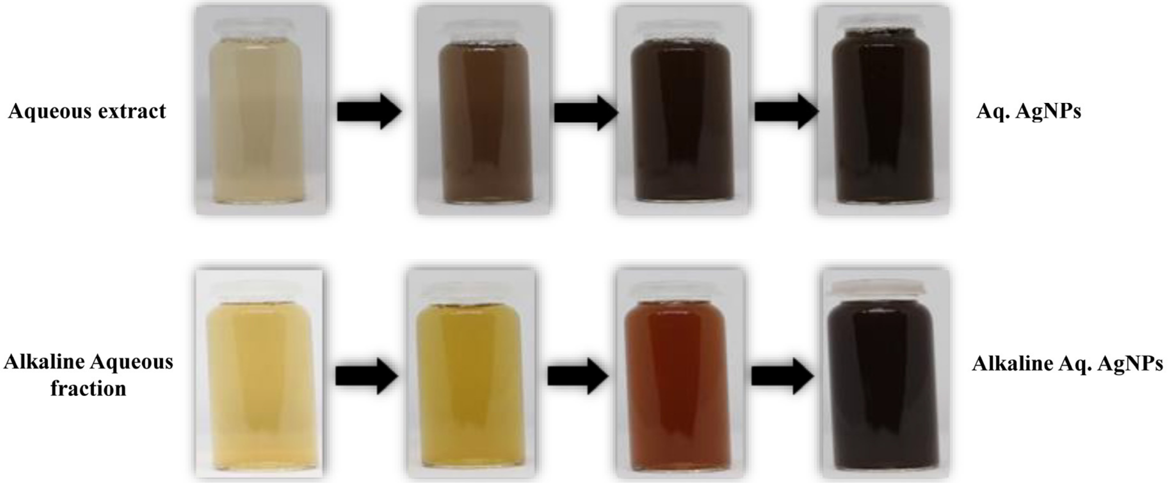

Figure S1: Color change of the AgNPs biosynthesis process by *R. stricta* Aq-extract and its alkaline fraction
